# BDNF-TrkB signaling through Erk1/2^MAPK^ phosphorylation mediates the enhancement of fear memory induced by glucocorticoids

**DOI:** 10.1038/mp.2013.134

**Published:** 2013-10-15

**Authors:** J-M Revest, A Le Roux, V Roullot-Lacarrière, N Kaouane, M Vallée, F Kasanetz, F Rougé-Pont, F Tronche, A Desmedt, P V Piazza

**Affiliations:** 1 INSERM U862, Neurocentre Magendie, Pathophysiology of Addiction, Bordeaux, France; 2 Pathophysiology of Neuronal Plasticity, Université de Bordeaux, Bordeaux, France; 3 INSERM U862, Neurocentre Magendie, Pathophysiology of Declarative Memory, Bordeaux, France; 4 CNRS UMR7224, UPMC Université Pierre et Marie Curie, Molecular Genetics, Neurophysiology and Behavior, Paris, France

**Keywords:** brain-derived neurotrophic factor, contextual fear memory, extracellular signal-regulated kinases 1/2 mitogen-activated protein kinase, glucocorticoids, tissue plasminogen activator, tropomyosin-related kinase B

## Abstract

Activation of glucocorticoid receptors (GR) by glucocorticoid hormones (GC) enhances contextual fear memories through the activation of the Erk1/2^MAPK^ signaling pathway. However, the molecular mechanism mediating this effect of GC remains unknown. Here we used complementary molecular and behavioral approaches in mice and rats and in genetically modified mice in which the GR was conditionally deleted (GR*^NesCre^*). We identified the tPA-BDNF-TrkB signaling pathway as the upstream molecular effectors of GR-mediated phosphorylation of Erk1/2^MAPK^ responsible for the enhancement of contextual fear memory. These findings complete our knowledge of the molecular cascade through which GC enhance contextual fear memory and highlight the role of tPA-BDNF-TrkB-Erk1/2^MAPK^ signaling pathways as one of the core effectors of stress-related effects of GC.

## Introduction

Glucocorticoids (GC) are adrenally secreted steroid hormones that are central in mediating the behavioral consequences of stress.^1, 2, 3, 4^ While, acute stress-induced high levels of GC increases the memory of stress-associated events,^1, 5, 6, 7, 8^ sustained GC secretion, induced by chronic stress, may lead to behavioral pathologies such as depression, anxiety, drug abuse and post-traumatic stress disorders.^1, 2, 3, 4, 5, 9, 10^ The majority of the GC's behavioral effects involve the activation of the ubiquitously neural-expressed glucocorticoid receptors (GR).^11, 12^ GR are GC-activated transcription factors that by multiple mechanisms lastly modify protein expressions.^12^ Consequently, identifying the molecular targets of GC-activated GR appears to be crucial to the understanding of the molecular mechanisms by which environmental changes can influence the activity of the central nervous system and induce behavioral impairments.^1^

In previous papers, we described a GR-induced molecular pathway (GR_Egr-1_MAPK_Syn-Ia/Ib; GEMS) occurring within the hippocampus that allows the enhancement of contextual fear memory.^7, 8^ These studies emphasized that the activation of the mitogen-activated protein kinase (MAPK) pathway,^13^ and in particular of Erk1/2^MAPK^ phosphorylation, is crucial in mediating the behavioral effects of GC. Thus GC, through the GR, activate the Erk1/2^MAPK^ signaling pathway via two parallel but independent mechanisms.^7^ The first is a direct transcriptional enhancement of the major proteins of the Erk1/2^MAPK^ pathway and of the downstream transcription factor Egr-1. The second is the activation by phosphorylation of Erk1/2^MAPK^ proteins that prolongs the increase in Egr-1 expression.^7^ Phosphorylated Erk1/2^MAPK^ in concert with Egr-1 modifies Synapsin-Ia/Ib protein expression and phosphorylation allowing the release of synaptic vesicles bound to actin.^8^ GR-induced phosphorylation of the Erk1/2^MAPK^ proteins is a necessary step for the GR-induced increase in contextual fear memory, as this behavior does not occur if Erk1/2^MAPK^ signaling is blocked.^7^ Unfortunately, the molecular mechanism of GR-induced Erk1/2^MAPK^ phosphorylation remains unknown. The understanding of this key step in the GEMS pathway is crucial to identify new molecular targets that will make it possible to design innovative therapies for stress-related disorders.

In this report, we addressed this issue by analyzing the involvement of the neurotrophic molecule BDNF (brain-derived neurotrophic factor) as an intermediate factor mediating the GC's effects on the induction of Erk1/2^MAPK^ phosphorylation. BDNF was chosen as a target for several reasons. First, both hippocampal BDNF mRNA production and GR activation are observed after acute stress and during contextual fear conditioning; two behavioral procedures that largely depend on glucocorticoid hormones.^10, 14, 15, 16, 17, 18, 19^ Second, BDNF is a major molecular player in the regulation of memory processes and related physiological functions such as synapse formations and synaptic plasticity.^20, 21, 22, 23^ For example, invalidation of the *BDNF* gene induced memory deficits in a context-dependent fear-conditioning procedure.^22, 24^ Third, the cellular effects of BDNF, mediated by the activation of the TrkB (tropomyosin-related kinase B) receptor,^23, 25, 26^ in many cases involve the activation of the Erk1/2^MAPK^ pathway.^23, 27^ In line with this observation, TrkB knockout mice and transgenic mice overexpressing TrkB have reduced and enhanced hippocampal-dependent memory, respectively.^28, 29^

The expression and activity of BDNF-TrkB and Erk1/2^MAPK^ signaling pathways in response to GC were studied using hippocampal extracts of corticosterone-treated mice and rats and of GR genetically modified mice (GR^*NesCre*^) in which the expression of GR was conditionally suppressed.^7, 12, 30^ Functional involvement of both BDNF-TrkB and Erk1/2^MAPK^ signaling pathways and BDNF proteolytic processing in fear-related memories were investigated using the inhibitors of these molecular pathways: TrkBFc, UO126 and PAI-1 (plasminogen activator inhibitor-1), respectively.^7, 31, 32^

Our results show that GR activation by stress-induced GC secretion increases the expression of both pro-BDNF and tPA (tissue plasminogen activator) proteins. GR-induced tPA, the role of which is to cleave plasminogen to plasmin, allows the proteolytic processing of pro-BDNF in mature BDNF increasing BDNF levels during stress. Mature BDNF then binds to TrkB receptor inducing its phosphorylation, which in turn phosphorylates Erk1/2^MAPK^ proteins that trigger the molecular cascade enhancing fear memories. These results thus identify GR-induced enhancement of hippocampal tPA-BDNF-TrkB signaling as the upstream molecular effectors of Erk1/2^MAPK^ phosphorylation, which mediate the enhancement of contextual fear memory induced by GC.

## Materials and methods

### Chemicals

Corticosterone was used at 10 nM on rat hippocampal slices and infused at 10 ng per hemisphere within the hippocampus. In all the experiments, we used a preformed water-soluble complex of corticosterone and 2-hydroxypropyl-β-cyclodextrin (#C174, Sigma, St Louis, MO, USA). MEK1/2 inhibitor; UO126 (#9903, 5 μM per hemisphere, Cell Signaling Technology, Danvers, MA, USA) and TrkBFc (#T8694; 390 ng per hemisphere, Sigma) were used to block Erk1/2^MAPK^ and BDNF signaling pathways, respectively.^7, 31^ Millipore (Billerica, MA, USA) provided the recombinant human BDNF (#GF029, 60 ng per hemisphere) and tPA inhibitor; stable recombinant mutant of human plasminogen activator inhibitor-1 (PAI-1; #528208, 30 ng per hemisphere).^31, 32^

### Hippocampal slice preparations and corticosterone treatment

A detailed description of hippocampal slice preparations has been described previously.^33^ Briefly, adult male Sprague-Dawley rats (aged 2–3 months, Charles River Laboratory, L'Arbresle, France) were used. Rats were then anesthetized with isoflurane and transcardially perfused with nearly frozen modified artificial cerebrospinal fluid (Csf) with 3 mM kynurenic acid. The modified Csf for perfusion contained: (in mM) 87 NaCl, 75 sucrose, 25 glucose, 5 KCl, 21 MgCl_2_, 0.5 CaCl_2_ and 1.25 NaH_2_PO_4_. After perfusion, brains were quickly removed and sliced (300 μm) in the coronal plane using a vibratome (Campden Instruments, UK). Immediately after cutting, slices were stored for 40 min at 32 °C in Csf ((in mM): 130 NaCl, 11 glucose, 2.5 KCl, 2.4 MgCl_2_, 1.2 CaCl_2_, 23 NaHCO_3_, 1.2 NaH_2_PO_4_), equilibrated with 95% O_2_/5% CO_2_ then stored at room temperature for the rest of the experiment. Each brain slice was then treated for 1 h and 3 h with 10 nM of corticosterone. One slice served as a control reference and did not undergo any treatment. Dorsal hippocampi were isolated, and proteins were extracted as previously described.^8^

### *In vivo* study of the interaction between GR, BDNF, TrkB, Erk1/2^MAPK^ and tPA

A detailed description was previously made elsewhere.^7, 8^ Briefly, for all the experiments, 4–6 month-old male C57/BL6J (Charles River Laboratory), GR^*LoxP/LoxP*^ and GR^*NesCre*^ mice were used. GR^*NesCre*^ mice display a conditional ablation of the GR gene (*Nr3c1*) only in the entire brain performed using the *Cre-LoxP* system.^30^ Experiments carried out in basal conditions compared control littermates GR^*LoxP/LoxP*^ (*n*=3) not expressing Cre and GR^*NesCre*^ (*n*=3) mice. For all the stress experiments, *n*=6 per group were used. GR^*NesCre*^, GR^*LoxP/LoxP*^ and C57/BL6J mice were subjected to a 30-min restraint stress and killed either in basal conditions (t0) or 30, 60 and 120 min after stress onset. Mice in the restraint-stressed group were placed in 50-ml conical centrifuge tubes (30 mm in diameter × 100 mm in length) fitted with a central puncture so as to allow ventilation. The tubes were placed in horizontal holders with light exposure.^19, 34^ At the end of the 30-min restraint procedure, the animals were killed, hippocampi and blood were collected and assayed for protein extraction and corticosterone assay, respectively. In experiments measuring the molecular effects of GC-mediated Erk1/2^MAPK^ signaling pathway enhancement, separate groups (*n*=6–9 per group) of C57/BL6J mice were killed 30 min after intra-hippocampal infusion of corticosterone (10 ng per side) or vehicle (Csf) in presence or absence of TrkBFc scavenger molecule, respectively.^31^ Hippocampi were then collected and assayed for immunoblotting analysis. Experiments were approved by the Aquitaine-Poitou Charentes ethical committee in strict compliance of the French and European Communities Council Directive (86/609/EEC).

### Protein extraction from brain tissues and immunoblotting analysis

A detailed description of protein extraction and immunoblotting analysis was previously reported.^7, 8, 19, 35^ Briefly, nuclear/cytoplasmic and total protein samples extracts from mouse and rat hippocampi were performed in RIPA buffer containing protease and phosphatase inhibitors (Sigma) before being subjected to immunoblotting experiments. SDS-PAGE-separated proteins were then revealed with relevant antibodies. Rabbit polyclonal antibodies anti-GR (#sc-1004-X; 1/20 000) and anti-BDNF (#sc-546; 1/1000) were from Santa Cruz Biotechnology (Santa Cruz, CA, USA), anti-PAI-1 (LSBio#C81062, 1/1000) was from Lifespan Biosciences (Seattle, WA, USA), anti-Erk1/2^MAPK^ (#06–182; 1/100 000) was from Millipore, anti-Phospho-Erk1/2^MAPK^ (#9101S; 1/1000) was from Cell Signaling Technology, anti-tPA (#T5600-05G; 1/2000) was from US Biologicals (Salem, MA, USA). Monoclonal antibodies anti-Phospho-TrkB (#2149-1; 1/5000) were from Epitomics (Burlingame, CA, USA), anti-TrkB (#610101; 1/2000) was from BD Biosciences (Franklin Lakes, NJ, USA), anti-Neuronal Class III β-Tubulin (TUJ1) (#MMS-435P; 1/20000) was from Eurogentec (Seraing, Belgium). In all experiments, βIII-tubulin measure was used as a loading control. X-Ray films (Kodak, Rochester, NJ, USA) were quantified by densitometry (optical density; OD) using a GS-800 scanner coupled with Quantity One software (Bio-Rad, Hercules, CA, USA).

### Corticosterone assay

Plasma corticosterone was quantified by radioimmunoassay using a specific corticosterone antibody (ICN Pharmaceuticals, Costa Mesa, CA, USA) as described elsewhere.^19, 35^

### Behavioral experiments.

#### Subjects and surgery

Male C57/BL6J mice (*n*=80 in Experiment 1*, n*=70 in Experiment 2 and *n*=70 in Experiment 3) 3–4 months old (Charles River Laboratory) were used and surgically implanted bilaterally 1 mm above the dorsal hippocampus, and then allowed to recover for 8 days before the behavioral experiments.^7, 8, 16^

#### Contextual fear-conditioning procedure

This behavioral procedure has been repeatedly used and fully described in previous studies.^7, 8, 16^ Briefly, each animal was placed in the conditioning chamber for 4 min during which it received two footshocks either 0.3 mA, 50 Hz, 3 s (L= low shock intensity group) or 0.7 mA, 50 Hz, 3 s (H= high shock intensity group), which never co-occurred with two tone (63 db, 1 KHz, 15 s) deliveries. Each animal returned to its home cage, and 24 h later the mice were re-exposed to the conditioning chamber. The freezing behavior, used as an index of conditioned fear, was calculated as a percentage (±s.e.m.) of the total time spent freezing during the first 2-min period of the retention test.

#### Microinjections

Immediately after acquisition of fear conditioning, animals were randomly divided into groups according to their intra-hippocampal infusion: Experiment 1: vehicle (Csf), corticosterone (10 ng per side, 0.5 μg μl^−1^), TrkBFc (390 ng per side, 1.3 μg μl^−1^), corticosterone+TrkBFc, BDNF (60 ng per side, 0.2 μg μl^−1^), BDNF+TrkBFc. Experiment 2: vehicle (2% DMSO in Csf), BDNF (60 ng per side), UO126 (5 μM per side), BDNF+UO126. Experiment 3: vehicle (Csf), corticosterone (10 ng per side, 0.5 μg μl^−1^), PAI-1 (30 ng per side, 0.1 μg μl^−1^), corticosterone+PAI-1, corticosterone+PAI-1+BDNF (60 ng per side, 0.2 μg μl^−1^). Infusions of 0.3 μl per hemisphere were performed at a constant rate (0.1 μl min^−1^). Corticosterone, TrkBFc, BDNF and PAI-1 were dissolved in Csf and UO126 in 2% DMSO in Csf.

#### Histology

A detailed description of the histological protocol was previously reported.^7, 8, 16^ Briefly, after completion of the behavioral study, animals were killed to evaluate the cannulae placements.

### Statistics

All values are given as mean±s.e.m. Statistical analyses were performed using analysis of variance followed by either Newman-Keuls or Fisher's PLSD *post-hoc* test for pairwise comparisons. The Student's *t*-test was used for pairwise comparisons.

## Results

### Activation of the GR in the hippocampus is a necessary condition for stress-induced increase in pro-BDNF expression and in BDNF levels

In a first series of experiment, we analyzed if a physiological increase in GC levels would regulate the expression of BDNF in the hippocampus. For this purpose, we used restraint stress, that is, a procedure inducing a reliable increase in GC levels. In addition, we have recently shown that GC injections and restraint stress can interchangeably increase fear memories.^10^

More precisely, we studied the expression of BDNF and of its precursor pro-BDNF^36, 37^ in response to a restraint stress (30 min) in GR^*NesCre*^ mice, in which the expression of GR has been conditionally suppressed in the entire brain.^7, 8, 19, 30^ Hippocampal protein extracts were analyzed by western blot in basal condition (t0), immediately after stress (t30 min) and 2 h (t120 min) after stress onset.

**Figure 1:**
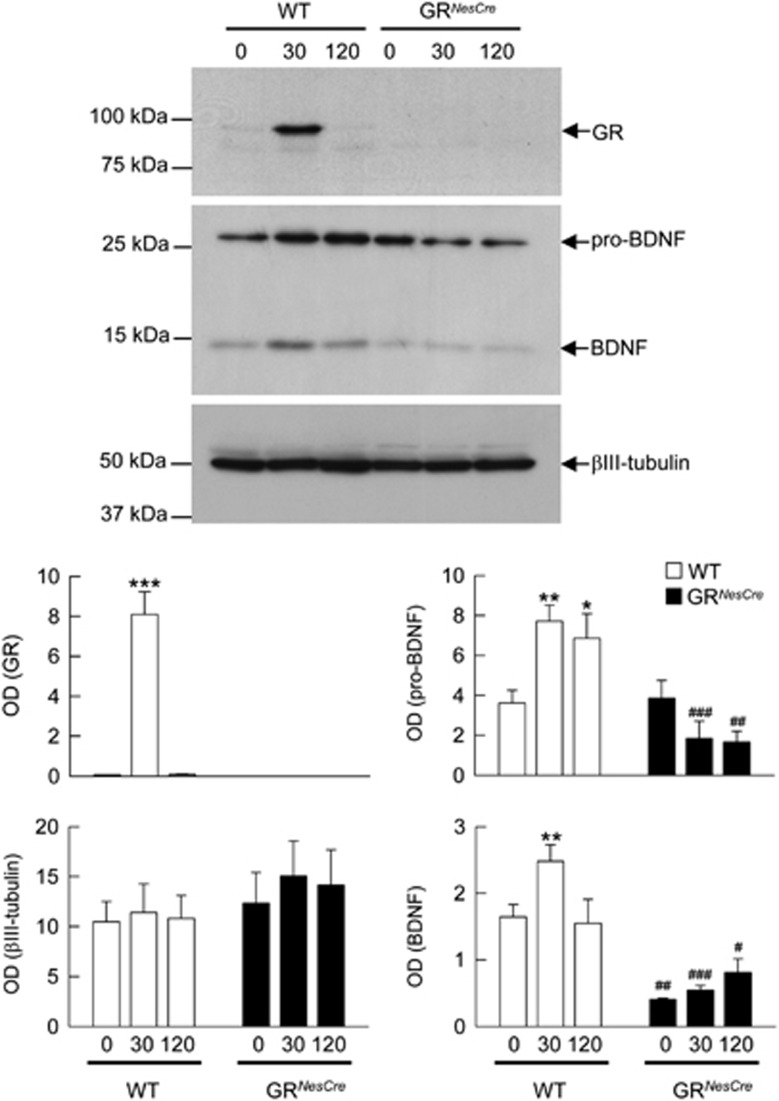
Stress-induced activation of the glucocorticoid receptor (GR) in the hippocampus stimulates pro-brain-derived neurotrophic factor (pro-BDNF) expression and its processing to mature BDNF. Comparison of the expression of pro-BDNF and BDNF proteins in wild-type (WT) and GR^*NesCre*^ mice, before (t0), 30 and 120 min after the onset of 30 min of restraint stress. Nuclear (for GR) and cytoplasmic hippocampal extracts were analyzed by western blot. X-Ray films were quantified by densitometry (OD). *P<0.05; **P<0.005, ***P<0.001 in comparison with t0 of each group. ^#^*P*<0.05, ^##^*P*<0.005, ^###^*P*<0.001 in comparison with the corresponding time point of WT. Newman-Keuls *post-hoc* test after analysis of variance.

In control littermate mice (WT), restraint stress induces translocation of the GR and increases the expression of pro-BDNF. This expression is maximal immediately after stress and is maintained 2 h later. BDNF levels increases 30 min after the beginning of the stress and returns to basal level after 2 h. In GR^*NesCre*^ mice, BDNF levels were reduced in basal conditions but no significant changes were observed in both pro-BDNF expression and in BDNF levels after stress, although a trend to decrease in pro-BDNF and to increase in BDNF were observed (Figure 1). This non-significant trend to increase in BDNF in GR^*NesCre*^ mice could correspond to a residual GR-independent proteolytic processing of the initial pool of pro-BDNF that consequently decreases in these mice, as its stress-induced increase is prevented by GR deletion.

Taken together, these results show that expression of the GR is a necessary condition for stress-induced increase in the production and processing of BDNF. As GR are the main molecular targets of stress-induced increase in GC, these results also suggest that stress-induced increase in GC through an activation of the GR upregulates pro-BDNF expression and BDNF levels.

### Activation of the GR in the hippocampus is a necessary condition for stress-induced increase in tPA expression

Processing of pro-BDNF into BDNF uses both intra- and extracellular enzymatic mechanisms that involve furin/proconvertases-like enzymes and plasmin, respectively.^36, 38, 39, 40, 41^ When pro-BDNF levels rapidly increase, the less efficient intracellular cleavage of furin/proconvertases-like enzymes leave most of the pro-BDNF protein uncleaved.^37, 42, 43^ Consequently, plasmin is principally responsible for processing secreted extracellular pro-BDNF, when the concentrations of this protein highly increase as in the case of stress.^38, 40, 41^

For these reasons, we studied if stress-induced GR activation controlled the proteolytic processing of pro-BDNF by the plasmin system. For this purpose, we focused on the enzyme tPA that cleaves plasminogen into plasmin, activating the enzymatic cascade that process pro-BDNF.^37, 43, 44^ tPA was a likely candidate also because this enzyme is activated after stress,^45^ after the injection of corticotropin-releasing factor, a critical component of the behavioral response to stress^46^ and has been involved in learning and memory.^47, 48^

**Figure 2:**
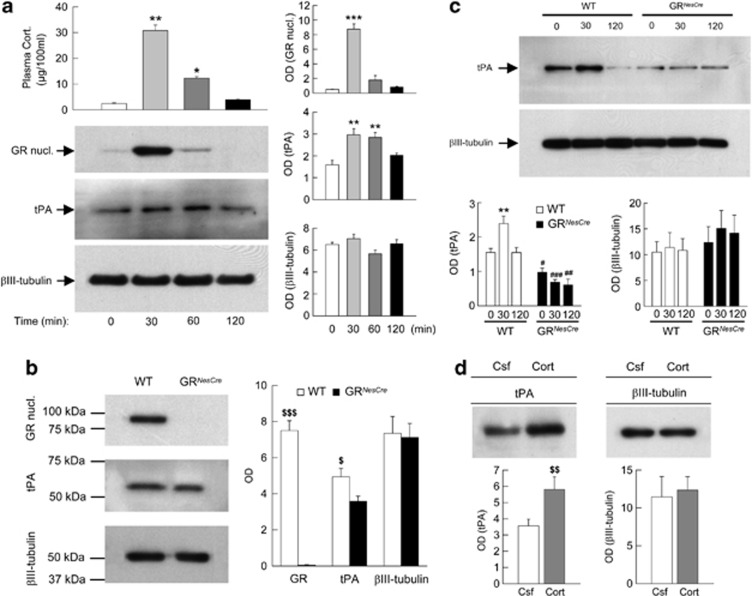
*In vivo* tPA expression in C57/Bl6J mice after stress (**a**), in GR^*NesCre*^ mice compared with control littermates in basal condition (**b**) and in response to restraint stress (**c**), and in C57/Bl6J mice after corticosterone injection (**d**). (**a**) Stress-induced glucocorticoid receptor (GR) activates tPA in the hippocampus. Plasma corticosterone concentrations, western blot and densitometric analyses of GR, tPA and βIII-tubulin proteins from cytoplasmic and nuclear hippocampal extracts of C57/Bl6J mice were measured before (t0) and 30, 60 and 120 min after the onset of 30-min restraint stress. (**b**) Western blot and densitometric analyses of GR, tPA and βIII-tubulin proteins from cytoplasmic and nuclear hippocampal extracts carried out in basal condition in wild-type (WT) and GR^*NesCre*^ mice. (**c**) Western blot and densitometric analyses of tPA and βIII-tubulin proteins from total hippocampal extracts of WT and GR^*NesCre*^ mice, before (t0), 30 and 120 min after the onset of 30 min of restraint stress. (**d**) Western blot and densitometric analyses of tPA and βIII-tubulin proteins from total hippocampal extracts collected 1 h post intra-hippocampal infusion of corticosterone (10 ng per side) in C57/Bl6J mice. Cort, corticosterone, Csf, cerebrospinal fluid. *P<0.05; **P<0.005, ***P<0.001 in comparison with t0 of each group. ^#^*P*<0.05, ^##^*P*<0.005, ^###^*P*<0.001 in comparison with the corresponding time point of WT. Newman-Keuls *post-hoc* test after analysis of variance. ^$^*P*<0.05, ^$$^*P*<0.005, ^$$$^*P*<0.001 in comparison with the matched control (WT or Csf groups). Student's *t*-test.

We first studied the effects of a restraint stress on the expression of tPA (Figure 2a). In C57/BL6J mice, 30 min of restraint stress increased plasma concentrations of corticosterone and transiently activated the GR, as indicated by the increase in the nuclear fraction of the GR 30 min after stress onset. Restraint stress also increased the expression of tPA that was maximal 30 min after stress, and still significantly elevated after 1 h (Figure 2a). We then analyzed tPA expression in GR^*NesCre*^ mutant mice lacking the GR. In basal conditions, tPA expression was significantly reduced in GR^*NesCre*^ mutant mice (Figure 2b). In addition, the increase in tPA observed in wild-type mice during stress was completely suppressed in GR^*NesCre*^ mutant mice (Figure 2c).

Taken together, these results indicate that the GR exert a tonic positive control on the expression of tPA (Figure 2b) as shown by the decrease in basal levels of tPA observed in GR^*NesCre*^ mutant mice. GR also appear as a necessary condition for the stress-induced increase in tPA expression (Figure 2c). As said before, GR are the main molecular targets of a stress-induced increase in GC. Thus, these results also suggest that stress-induced increase in GC through an activation of the GR upregulates tPA during stress.

The decrease of basal levels of tPA observed in GR^*NesCre*^ mice likely explains the decrease in basal levels of BDNF observed in these animals in the previous experiment (Figure 1). In contrast, the non-significant trend to a slow and moderate increase in BDNF and the parallel trend to decrease in pro-BDNF observed after stress in GR^*NesCre*^ mice could be mediated by a residual, GR-independent, activity of the low efficient furin-like enzymatic system.

To further investigate the role of GC, we analyzed if an acute administration of corticosterone could have a similar effect to the one of stress in regulating the expression of tPA. For this purpose, we studied the expression of tPA in C57/BL6J mice intra-hippocampally-injected with corticosterone (10 ng per side) and then killed it 1 h later. Intra-hippocampal corticosterone injection similar to what was observed after stress increased the expression of tPA (Figure 2d). It is possible that 10 ng per side of corticosterone transiently induce in the hippocampus higher corticosterone levels than the ones observed after stress. However, 30 min after the infusion of 10 ng per side of corticosterone, we found corticosterone concentrations (35 ng g^−1^) in the hippocampus (Desmedt *et al.*^16^, unpublished results) that were similar to the ones that can be expected in stress conditions (27 ng m^−1^l) based on the levels of free hippocampal corticosterone measured by Qian *et al.*^49^, using microdialysis. Therefore, these results extend the role of GC in tPA regulation, suggesting that these hormones could also be a sufficient condition for increasing tPA expression.

Altogether, the present results indicate that GC through activation of the GR exert tonic and phasic positive controls on the expression of the tPA protein in the hippocampus.

### GC-activated BDNF-TrkB signaling mediates the enhancement of contextual fear memory induced by glucocorticoids

The results of the previous experiments indicate that GC-activated GR control in a coordinate way the expression of pro-BDNF and tPA proteins, which ultimately results in the increase of BDNF. Part of the BDNF signaling effects are mediated by the activation of the TrkB receptor,^23, 25^ which among other effects is able to activate the Erk1/2^MAPK^ signaling pathway, inducing the phosphorylation of the Erk1/2^MAPK^ proteins.^23, 27^ Therefore, we studied whether upregulation of BDNF by GC also induces the activation of TrkB receptor and, in particular, TrkB phosphorylation.

**Figure 3:**
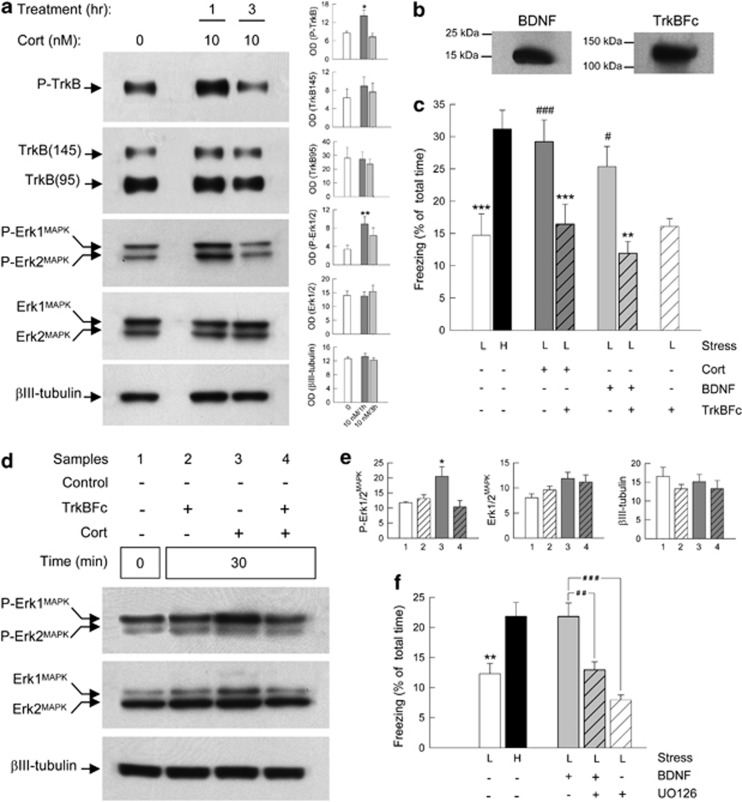
Enhancement of contextual fear memory by glucocorticoid (GC) occurring through BDNF-TrkB signaling requires Erk1/2^MAPK^ activation. (**a**) *Ex vivo* TrkB receptor phosphorylation is mediated by GC. Western blot and densitometric analyses of P-TrkB, 145-kDa and 95-kDa TrkB isoforms, P-Erk1/2^MAPK^, Erk1/2^MAPK^ and βIII-tubulin proteins from dorsal hippocampal slices extracts of Sprague-Dawley rats incubated with 10 nM corticosterone for 1 and 3 h. *P<0.05, **P<0.005, compared with control slices. (**b**) Western blot showing purified brain-derived neurotrophic factor (BDNF) and TrkBFc proteins injected into the hippocampus of C57/BL6J mice. (**c**) BDNF-TrkB signaling mediates the enhancement of contextual fear conditioning induced by GC. Percentage of freezing was measured in C57/BL6J mice for 2 min to the conditioning context 24 h after conditioning with either high (0.7 mA footshock: H, black bar) or low shock intensity (0.3 mA footshock: L, white bar) and receiving a post-training intra-hippocampal infusion of either corticosterone (dark gray bar), BDNF (light gray bar) with or without TrkBFc (striped bars). Fisher's PLSD *post-hoc* test after analysis of variance (ANOVA). ***P<0.001 compared with high and low+Cort, respectively. **P<0.005 compared with low+BDNF. ^#^*P*<0.05 and ^###^*P*<0.001 compared with low, respectively. (**d**) Western blot analysis and (**e**) quantification of hippocampal expression of P-Erk1/2^MAPK^, Erk1/2^MAPK^ and βIII-tubulin proteins in C57/Bl6J mice that were hippocampally infused with corticosterone (10 ng per side) for 30 min in presence or not of TrkBFc. Hippocampal proteins were analyzed by western blot and densitometrically quantified. *P<0.05 in comparison with all other groups, Newman-Keuls *post-hoc* test after ANOVA. (**f**) Enhancement of contextual fear memory by BDNF depends on Erk1/2^MAPK^ signaling. Percentage of freezing was measured in C57/BL6J mice for 2 min to the conditioning context 24 h after conditioning with either high (H, black bar) or low shock intensity (L, white bar) and receiving a post-training intra-hippocampal infusion of BDNF (light gray bar) with or without MEK1/2 inhibitor; UO126 (striped bars). Fisher's PLSD *post-hoc* test after ANOVA. **P<0.005 compared with high. ^##^*P*<0.005 and ^###^*P*<0.001 compared with low+BDNF+UO126 and low+UO126, respectively.

To address this issue, we first analyzed the activation of TrkB receptor in hippocampal slices treated with GC by measuring the phosphorylation level of the TrkB receptor. Hippocampal slices of rat brain were incubated for 1 h and 3 h in the presence of 10 nM of corticosterone and dorsal hippocampi were then analyzed by western blot. The results showed an increase of phosphorylated TrkB after 1 h of treatment with corticosterone and a concomitant increase in the phosphorylation of Erk1/2^MAPK^ (Figure 3a), confirming the involvement of the TrkB receptor in the molecular pathway activated by GC.

These results suggest that GC-induced increase in hippocampal TrkB activation by BDNF could mediate the enhancement of contextual fear memory induced by GC. If this hypothesis is true, preventing BDNF signaling should block the enhancement of fear memory induced by GC. To test this hypothesis, we studied the behavioral effects of an intra-hippocampal injection of the BDNF signaling inhibitor, TrkBFc, which is a soluble ‘scavenger' form of the TrkB receptor (Figure 3b).^31^ We used a contextual fear-conditioning procedure in mice that depends on the functional integrity of the hippocampus, on the level of stress and on GC-activated GR.^7, 8, 16, 19, 50^ Conditioned fear was measured in the conditioning context, 24 h after conditioning by measuring the freezing behavior of the mice. In this task, a high shock (H) intensity exerted during conditioning induced a higher level of conditioned fear than a low shock intensity (L) (Figure 3c). As previously shown, an injection of corticosterone immediately after conditioning with a low shock intensity (L+Cort) increased fear memory to the levels observed with an electric shock of high intensity (Figure 3c and Revest *et al.*^7, 8^). The enhancement of fear memory induced by GC were completely reversed by the concomitant intra-hippocampal infusion of TrkBFc (L+Cort+TrkBFc), suggesting that GC effects were mediated by BDNF. To prove this point, we analyzed if BDNF could substitute to corticosterone in increasing fear memory. This appeared to be the case, as infusion of BDNF in the hippocampus of animals conditioned with low shock intensity (L+BDNF) increased fear memory to an extent similar to the one observed after corticosterone infusion (L+Cort, Figure 3c). As expected, these effects of BDNF were blocked by TrkBFc infusion (L+BDNF+TrkBFc, Figures 3b and c).

These results indicate that the enhancement of contextual fear memory mediated by GC involves the activation of the TrkB receptor by BDNF.

### GC-induced BDNF-TrkB signaling enhances fear conditioning via Erk1/2^MAPK^ signaling

The previous results provided evidence that GC activate in parallel the BDNF-TrkB and Erk1/2^MAPK^ signaling pathways (Figure 3a) and that similarly to what was previously shown for Erk1/2^MAPK^,^7^ BDNF-TrkB activation mediates the fear-related behavioral effects of GC (Figure 3c). However, these results do not elucidate whether the activation of the Erk1/2^MAPK^ pathway induced by GC is a molecular process that is upstream or downstream to the BDNF-TrkB signaling. Thus, Erk1/2^MAPK^ phosphorylation could depend on the activation of TrkB induced by BDNF-dependent dimerization. Alternatively, the TrkB receptor, independently of BDNF binding, could be directly transactivated by Erk1/2^MAPK^ acting on the kinase domain of the TrkB receptor, a mechanism that has already been observed for other tyrosine kinase receptor.^51, 52, 53^ To clarify this issue, we first analyzed the effects of TrkBFc on the phosphorylation of Erk1/2^MAPK^ induced by GC. C57/BL6J mice received an intra-hippocampal infusion of corticosterone with or without TrkBFc.^31^ The mice were killed 30 min after the hippocampal injections and hippocampal proteins were analyzed by western blot. We found that the increase in Erk1/2^MAPK^ phosphorylation induced by GC infusion was largely abolished by TrkBFc (Figures 3d and e). These results then suggest that GC-induced Erk1/2^MAPK^ phosphorylation is a process that requires TrkB activation and is consequently localized downstream of the activation of BDNF-TrkB signaling (Figures 3d and e). To further confirm this sequence of molecular events at a behavioral level, we analyzed if the increase in fear memory induced by BDNF infusion, observed in the previous experiment (Figure 3c), could be abolished if Erk1/2^MAPK^ phosphorylation was prevented.

For this purpose, we studied the behavioral effects of an intra-hippocampal injection of the MEK inhibitor UO126 on BDNF-induced enhancement of fear memories.^7^ As found in the previous experiment (Figure 3c), injection of BDNF in low shock condition (L+BDNF) increased fear conditioning mimicking the effects of a shock of high intensity (H) (Figure 3f). This increase in fear memory induced by BDNF was abolished by the injection, immediately after conditioning, of UO126 (L+BDNF+UO126).

In conclusion, together with previous experiments that showed that GC increase fear memory through the activation of the Erk1/2^MAPK^ cascade,^7^ the present results demonstrate that the GC-mediated enhancement of contextual fear memory occurs through the activation of the TrkB receptor by BDNF, which in turn activates downstream the Erk1/2^MAPK^ cascade.

### tPA-mediated pro-BDNF proteolytic processing is required for the enhancement of GC-induced contextual fear memory

**Figure 4:**
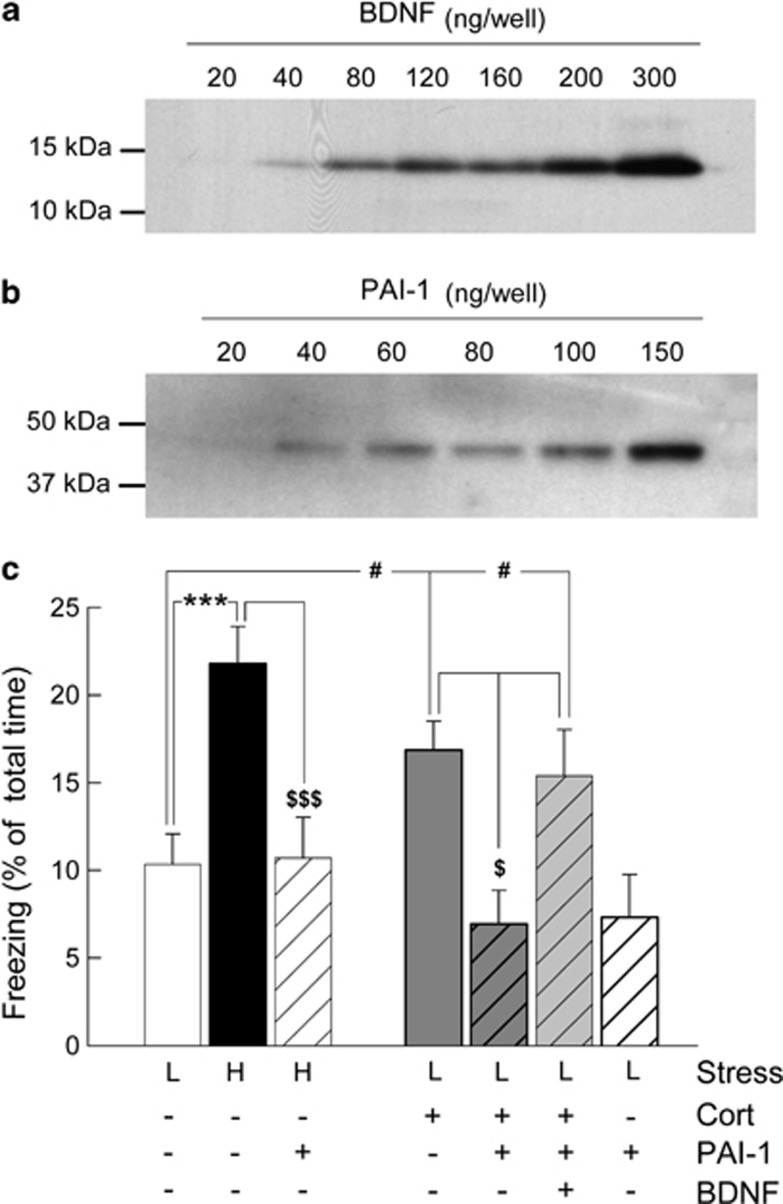
Enhancement of contextual fear memory induced by glucocorticoid (GC) requires pro-brain-derived neurotrophic factor (pro-BDNF) proteolytic processing by tPA/Plasmin system. Western blot showing purified (**a**) BDNF and (**b**) PAI-1 proteins injected into the hippocampus of C57/BL6J mice. (**c**) Percentage of freezing was measured for 2 min to the conditioning context 24 h after conditioning with either high (H, black bar) or low shock intensity (L, white bar) and receiving a post-training intra-hippocampal infusion of either corticosterone (dark gray bar), corticosterone+BDNF (light gray bar) with or without tPA inhibitor; PAI-1 (striped bars). Fisher's PLSD *post-hoc* test after analysis of variance. ***P<0.001 compared with high. ^$$$^*P*<0.001 and ^$^*P*<0.05 compared with high and low+Cort and low+Cort+PAI-1+BDNF (striped light gray bar), respectively. ^#^*P*<0.05 compared with low+Cort and low+Cort+PAI-1+BDNF, respectively.

The results of the previous experiments indicated that GC- and stress-induced activation of GR increase tPA expression (Figure 2) and BDNF-TrkB molecular signaling, which in turn induce Erk1/2^MAPK^ phoshorylation to finally enhance contextual fear memory (Figures 1 and 3). However, these results do not demonstrate that the enhancing effects of GC on contextual fear memory occurring through the activation of the BDNF-TrkB-Erk1/2^MAPK^ signaling cascade require the activity of tPA to cleave pro-BDNF in BDNF. To test this hypothesis, we analyzed if the inhibition of the activity of tPA would block the enhancement of contextual fear memory mediated by the GC-activated GR-BDNF-TrkB-Erk1/2^MAPK^ signaling cascade. For this purpose, we used the tPA inhibitor PAI-1 (plasminogen activator inhibitor-1)^32^ to prevent pro-BDNF proteolytic processing into mature BDNF. The infusion of PAI-1 in the hippocampus, immediately after conditioning, blocked the increase in fear conditioning induced by a shock of high intensity (H+PAI-1, Figures 4b and c) or by the infusion of corticosterone after a shock of low intensity (L+Cort+PAI-1, Figures 4b and c). The effects of PAI-1 were mediated by the inhibition of the production of mature BDNF, as PAI-1-induced inhibition of fear memory was rescued by the concomitant infusion of mature BDNF (L+Cort+PAI-1+BDNF, Figures 4a–c).

These results indicate that the enhancement of fear memory by GC occurs through tPA-induced pro-BDNF proteolytic processing that by increasing BDNF levels activates the TrkB-Erk1/2^MAPK^ signaling cascade.

## Discussion

**Figure 5:**
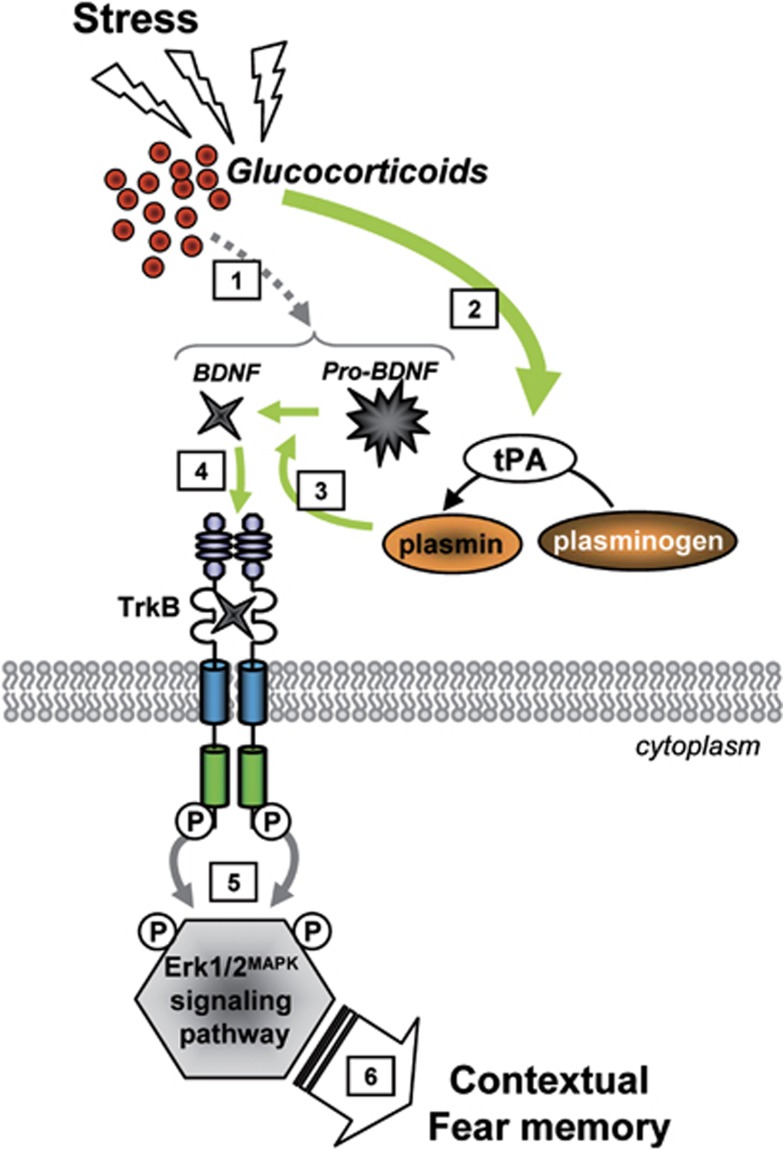
Schematic model of glucocorticoid (GC)-induced molecular mechanism mediating the enhancement of contextual fear memory. Stress-increased GC secretion induces the expression of pro-brain-derived neurotrophic factor (pro-BDNF) (1) and tPA (2) proteins. tPA, whose role is to cleave plasminogen to plasmin, allows proteolytic processing of pro-BDNF into BDNF (3). Mature BDNF binds to and activates TrkB receptor (4). TrkB phosphorylation induces further phosphorylation and activation of Erk1/2^MAPK^ signaling pathway (5) to mediate the enhancement of contextual fear memory (6). This model, based on several studies including our own, is discussed in the main text.

Taken together, the results of the present experiments complete the understanding of the molecular mechanism of fear-related behavioral effects of GR activation by GC, identifying the most upstream molecular effectors of the GEMS cascade.^7, 8^ In particular, they show that the enhancement of fear memory mediated by GR-induced Erk1/2^MAPK^ phosphorylation^7^ depends on the activation of the tPA-BDNF-TrkB signaling cascade. Using various *ex vivo* and *in vivo* molecular and behavioral approaches, we found that stress-induced activation of the GR stimulates pro-BDNF and tPA proteins, which in concert induce the increase in mature BDNF. The GR-mediated increase in BDNF, through the activation of the TrkB receptor, induces Erk1/2^MAPK^ phosphorylation, which finally results in an enhancement of fear memory (Figure 5).^7^ Indeed, preventing hippocampal BDNF-TrkB activation by the TrkBFc scavenger receptor abolished both GC-mediated increase in Erk1/2^MAPK^ phosphorylation and in contextual fear memory. In addition, the increase in conditioned fear induced by BDNF was also blocked by the MEK inhibitor UO126. Lastly, inhibition of tPA function by PAI-1 blocked the enhancement of fear memories induced by GC, an effect that was rescued by the concomitant infusion of mature BDNF.

The experiment performed here using GR genetically invalidated mice clearly show that GC-induced activation of the GR is a necessary condition for the activation of the tPA-BDNF-TrkB-Erk1/2^MAPK^ molecular cascade that leads to an increase in fear memories. However, this does not exclude that some of the stress-related effects of GC could also involve other mechanisms. For example, GC also activate the high-affinity mineralocorticoid receptors (MR). MR are transcription factors that are already fully activated for low basal levels of GC.^54^ Recent evidences using both pharmacological and genetic approaches targeting the MR indicate that these receptors are also able to regulate contextual fear memory^55^ with a specific role in retrieval of emotional information.^56^ Some of the effects of GC could also be mediated by cell adhesion molecules (CAMs), such as the polysialylated form of the neural cell adhesion molecule (PSA-NCAM)^57, 58^ or nectin-1,^59^ catecholamines in particular the stress hormone norepinephrine,^60^ or interleukins^61^ that also contribute to the modulation of contextual fear memory.

Although our data and previously published report^17, 18^ show that stress and GC increase BDNF levels, other reports have shown that when a longer stress is used (from 2 h to 8 h in comparison with 30 min used in our experiments), a suppression of BDNF expression is observed in specific subfields of the hippocampus (CA1 and CA3).^62, 63^ Taken together, these data suggest that the shift from a facilitating effect on memory of a short-lasting stress to a deleterious one induced by prolonged stress could be mediated by a fine-switch mechanism enhancing or decreasing BDNF levels, respectively. These potential opposite effects of GC on BDNF levels could be the reflection of the complexity of the molecular processing of BDNF both at a transcriptional and translational level. Thus, the *BDNF* gene is constituted by eight distinct promoters encoding nine exons that can undergo alternative splicing^64, 65, 66^ with the mutual existence of two polyadenylation sites leading to two populations of mRNA with specific and different subcellular localizations.^66, 67^ In addition, as said before, the BDNF protein is initially synthesized as a pro-BDNF, which is then cleaved in mature BDNF.^40, 42, 43^ Many data have now shown that pro-BDNF and BDNF display distinct and often opposed biological functions,^36, 68, 69^ confirming the value of working at the protein level and especially of studying the molecular mechanisms underlying the BDNF proteolytic processing. An oscillation in the balance between pro-BDNF and BDNF levels could have profound physiological implications. The conversion of pro-BDNF in mature BDNF by the tPA/plasmin system^40^ could facilitate adaptation to acute stress. In contrast, during chronic stress, the sustained secretion of GC, by a molecular mechanism that still needs to be precisely determined, could induce a decrease in BDNF mRNA expression^62, 70^ and the inhibition of the proteolytic conversion of pro-BDNF to mature BDNF. Given that pro-BDNF preferentially activates p75^NTR^ instead of the TrkB receptors, pro-BDNF could orientate cell fate toward apoptosis or synaptic plasticity toward LTD impairing memory.^68, 69, 71, 72^ Consistently with this hypothesis, p75^NTR^ knockout mice have enhanced spatial memory and hippocampal LTP.^73^ Our results also complete previous knowledge by providing the physiological context: enhancement of stress-related memory in which the coordinated activation of tPA-BDNF-TrkB-Erk1/2^MAPK^ signaling by the GR increases cognitive functions. Thus, several previous report have shown that the tPA/plasmin system,^40, 47, 48, 74, 75, 76, 77^ mature BDNF,^22, 24, 78, 79, 80, 81, 82^ and the TrkB receptor^23, 28, 29, 83^ *per se* can increase hippocampal-dependent memory and promote memory-related synaptic changes such as LTP.^83, 84^

The results of this paper and of our previous works^7, 8^ then reveal that stress-activated GR are the triggering and coordinating factor of a cascade of complementary molecular events that finally lead to an increase in fear memory. The starting point is a GR-induced rapid increase in the expression of pro-BDNF and tPA proteins and of the proteins of the Erk1/2^MAPK^ pathway. These events are followed by the phosphorylation of Erk1/2^MAPK^, which is mediated by GC-mediated activation of BDNF-TrkB signaling. The resulting sustained increase in phoshorylated Erk1/2^MAPK^ triggers the synthesis of the transcription factor Egr-1 that stimulates the transcription of synapsin-I, which increases the number of synaptic vesicles bound to actin and in this way the pool of readily releasable neurotransmitters.^8, 85^ Phosphorylated Erk1/2^MAPK^ induces in turn the phosphorylation of synapsin-I, which release synaptic vesicles bound to actin allowing their transport to the presynaptic membrane. This last step of the GEMS molecular pathway finally controls the release of excitatory neurotransmitters such as glutamate,^86, 87, 88, 89^ a modification in neural activity consistent with an increase in memory encoding.^90^

The knowledge provided here of the molecular cascade involved in stress-related effects of GC can help to improve our understanding of the pathophysiology of psychiatric diseases such as depression and post-traumatic stress disorders, in which a deregulation of GC and BDNF has been separately involved.^1, 2, 3, 4, 5, 9, 10^ For example, among biological markers that characterize depression, hypercortisolemia and a dysregulation of the hypothalamic-pituitary-adrenal (HPA) axis have been repeatedly found.^91^ In addition, even if BDNF genetically modified mice did not show alterations in depressive-like behaviors, they consistently displayed an inability to respond to antidepressant treatment^92^ Concerning post-traumatic stress disorder, GC^10^ and BDNF^92, 93^ have also been involved in the establishing the pathological form of traumatic memory that characterizes this disease. In this respect, beyond the study of GC and BDNF *per se*, the general analysis of post-translational modifications of the coordinated actions of these factors within the GEMS cascade could open new insight in the understanding of the molecular mechanisms of these two diseases. In particular, the understanding of the mechanisms through which GC-induced tPA control the pro-BDNF/BDNF balance could be of pathophysiological relevance.

In conclusion, the results provided here, by defining one of the molecular pathway involved in stress-related effects of GC can help developing new targeted treatments for several psychiatric diseases.
